# Jinn Possession; a Case Report Exploring Symptom Formation in Trans-Cultural Psychiatry

**DOI:** 10.1192/bjo.2025.10783

**Published:** 2025-06-20

**Authors:** Georgina Edgerley-Harris, Mohammad Arbabi, Nilesh Tirbhowan

**Affiliations:** 1 Surrey and Borders Partnership NHS Foundation Trust, Leatherhead, United Kingdom; 2 Sussex Partnership NHS Foundation Trust, Brighton, United Kingdom

## Abstract

**Aims:** We report on an Afghan refugee is in his 30s who presented to a Community Mental Health Recovery Service (CMHRS) with a two year history of visual and auditory disturbances and distressing persecutory beliefs revolving around 'Jinn'. This led to significant distress, a decline in functioning and shared experience with his wife also complaining of similar events.

**Methods:** Our patient’s symptoms commenced when he and his family moved into a new property and believed that his house was possessed by 'Jinn'. He had moved from Afghanistan to the UK as a refugee, speaking predominantly in Farsi and a practising Muslim. Examples of his experiences which he attributed to the ‘Jinn’ included; hearing noises in the house, seeing a broken cup and bathroom toiletries on the floor, and seeing a male figure during the night described as ‘frightening, headless and with claws’.

We conducted a thorough assessment and encompassed cultural and spiritual components of his health, liaising with their refugee support worker and involving our Trust multifaith team and a Farsi-speaking psychiatrist.

Affective conditions, Post Traumatic Stress Disorder and folie à deux were considered during the assessment period and felt that Adjustment Disorder reflected the current presentation. Precipitating factors included moving to UK, change in culture, and lack of local provisions to accommodate his faith such as no local access to a Mosque or Halal food shops.

Psychotropic medication was not indicated and the patient declined psychological therapy. Following movement of housing to an Afghan community, there were no further concerns expressed in relation to Jinn. The patient was subsequently discharged back to their GP.

**Results:** This case highlights the important influence religion and culture can have in symptom formation and how altered perceptions can be interpreted subjectively. The patient’s distress of migrating, separation from family and friends and change of cultural environment are likely to contribute to the patient’s experiences.

Symptoms of possession can overlap with mental illnesses and can be a socially accepted explanatory model of disease in non-Western cultures in expressing distress and conveying conditions including depression, bipolar disorder and anxiety along with stress and marital hardship.

**Conclusion:** Our patient’s diagnosis of Adjustment disorder highlights the importance of a holistic approach to psychiatric assessment. A better understanding of symptom formation within trans-cultural psychiatry could minimise the risks of subsequent misdiagnosis and inappropriate medication prescription. Correct formulation means we can support patients in accessing appropriate religious and spiritual resources and care where appropriate.

